# Dosage Sensitivity Shapes the Evolution of Copy-Number Varied Regions

**DOI:** 10.1371/journal.pone.0009474

**Published:** 2010-03-10

**Authors:** Benjamin Schuster-Böckler, Donald Conrad, Alex Bateman

**Affiliations:** 1 European Molecular Biology Laboratory-Centre for Genomic Regulation Systems Biology Unit, Centre for Genomic Regulation, Barcelona, Spain; 2 Wellcome Trust Sanger Institute, Wellcome Trust Genome Campus, Cambridge, United Kingdom; University of California, Riverside, United States of America

## Abstract

Dosage sensitivity is an important evolutionary force which impacts on gene dispensability and duplicability. The newly available data on human copy-number variation (CNV) allow an analysis of the most recent and ongoing evolution. Provided that heterozygous gene deletions and duplications actually change gene dosage, we expect to observe negative selection against CNVs encompassing dosage sensitive genes. In this study, we make use of several sources of population genetic data to identify selection on structural variations of dosage sensitive genes. We show that CNVs can directly affect expression levels of contained genes. We find that genes encoding members of protein complexes exhibit limited expression variation and overlap significantly with a manually derived set of dosage sensitive genes. We show that complexes and other dosage sensitive genes are underrepresented in CNV regions, with a particular bias against frequent variations and duplications. These results suggest that dosage sensitivity is a significant force of negative selection on regions of copy-number variation.

## Introduction

It has been estimated that at least 2% of the human genome is affected by structural variations [1], such as inversions, small insertions/deletions or large copy-number variants (CNVs) [2]. These sometimes large rearrangements can be seen as an important driving force of genome evolution [3]. As a consequence, theories on gene evolution have to be re-evaluated in the context of such rapid and widespread large scale variation. Previous studies have already shown that the locations and functional annotations of genes in CNV regions are strongly biased [1], [4]. CNVs are found more often in pericentromeric and subtelomeric regions and they overlap significantly with regions of segmental duplications. Genes within CNV regions are frequently involved in sensory perception and immune system activity, to a lesser extent in cell adhesion and in a number of cases signal transduction [1]. Furthermore, it has also been observed that copy-number variability is negatively correlated with protein interaction network metrics such as connectivity and centrality [5]. Two theories have been postulated to explain this non-random distribution of CNVs. The mutational hypothesis states that most CNVs are in effect phenotypically neutral, but are carried by flanking genomic elements like segmental duplication or ALU repeats which cause the bias in CNV distribution. The opposing theory could be called the selection hypothesis, stating that negative and positive selection shape the distribution of CNVs through the functional elements they encompass.

Gene duplication and loss are key mechanisms in evolution [6]. Historically, it was assumed in this context that most genes can be duplicated without substantial negative fitness effects. Similarly, the established hypothesis explaining gene dominance formulated by Wright [7] states that dominance is caused by “bottlenecks” in metabolic pathways and is generally rare [8]. This is in stark contrast to the observation that at least 20% of the entries in the OMIM database of human diseases with a Mendelian pattern of inheritance are described as heterozygous mutations [9]. It has also been shown that there are distinct differences between genes as to their duplicability [10], [11] and that duplicated genes are in many cases still under negative selection [12], [13]. Birchler *et al.* [14] reported widespread dosage compensation upon polyploidization of several large chromosomal regions in maize. For all these reasons, it is now widely accepted that some genes are dosage sensitive.

What are the underlying causes of dosage sensitivity? Papp *et al.* [15] postulated that multi-protein complexes need to maintain the stoichiometry of their subunits to perform their biological function (the balance hypothesis). A range of experiments lend support to the balance hypothesis. It has been noted that expression levels of interacting proteins are highly co-ordinated [16], hinting that proportionality of subunit abundances is important. In a previous study, we also reported an enrichment for dominant disease mutations amongst interacting proteins [17]. Within the conceptual framework of the balance hypothesis, this can be explained by the impact of even small stoichiometric changes (the one mutated allele) on the function of the entire protein complex. It has also been argued that tolerance towards polyploidization, compared to the sometimes severe effects of smaller duplications can be explained by conservation of stoichiometry [18]. Finally, it has been noted that highly-interacting proteins in higher organisms belong to small gene families [10], which could be conveniently explained by a bias against duplication acting on multi-protein complexes.

There have been, however, several conflicting reports. Deutschbauer *et al.* [19] performed an exhaustive heterozygous deletion screen in yeast. They reported only 3% of genes to be haploinsufficient. While these genes were enriched for members of protein complexes, their overexpression did not cause a similar phenotype as their deletion. Subsequently, Sopko *et al.* [20] systematically induced gene overexpression for all ORFs in yeast. The genes found to be toxic when overexpressed did not overlap with the haploinsufficient genes described by Deutschbauer *et al.*, and were not significantly enriched for protein complexes.

These findings point towards a more complex relationship between haploinsufficiency and duplication sensitivity [21]. A limited number of enzymes are sensitive to low dosage because they are the rate limiting factor in a biochemical reaction. A range of proteins are likely to cause non-physiological binding or even agglomeration as a result of overexpression, as exemplified by susceptibility to early-onset Alzheimer's disease as a result of duplication of the APP locus [22]. Finally, haploinsufficiency as well as duplication sensitivity are likely to affect those master-regulators controlling the balanced expression of a range of other proteins [23], [24]. These proteins are in fact often complexes [25].

The newly developed CORUM database [26] contains mammalian protein complexes that were manually annotated by expert curators. It contains a large number of gene regulatory and transcriptional genes, as listed in Table 1. In this work, we use gene expression and copy-number variation data to assess the relationship between protein complexes from CORUM, dosage sensitivity and recent gene evolution in the human population. We show that changes in gene copy number have a weak but measurable effect on gene expression. We find that protein complex genes are enriched for known dosage sensitive genes and exhibit substantially lower expressional noise than other genes. Consequentially, we observe that dosage sensitive genes are underrepresented in CNV regions.

**Table 1:** Composition of the CORUM database.

GO-Slim Term	Number of CORUM genes	P-Value
protein binding	1348	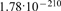
nucleus	1058	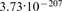
macromolecule metabolic process	1321	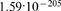
nucleobase, nucleoside, nucleotide and nucleic acid metabolic process	852	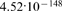
nucleic acid binding	708	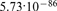
cytoplasm	933	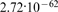
regulation of biological process	722	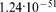
chromosome	168	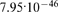
structural molecule activity	227	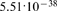
transcription regulator activity	301	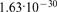
biosynthetic process	279	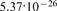
helicase activity	53	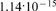
cell death	146	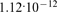
protein transporter activity	45	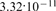
response to stimulus	378	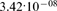
translation regulator activity	34	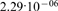
cell differentiation	232	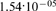
extracellular region	77	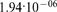
membrane	532	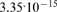

Underrepresented terms are set in bold font.

## Methods

### The CORUM Database of Mammalian Protein Complexes

**Figure 1:**
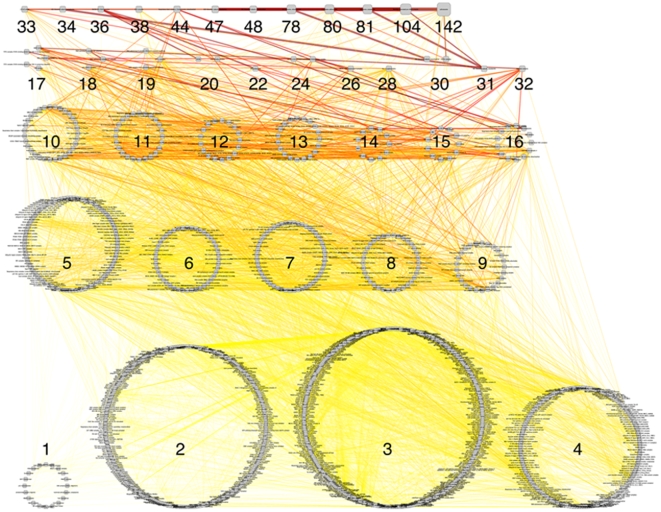
A network representation of the CORUM database. Nodes represent complexes and are ordered by number of unique components (shown as number next to groups). Edges denote shared components between complexes. The number of shared components is reflected in the colour (from yellow (few) to red (many) shared components) as well as in the line width. The large, highly overlapping complexes in the first row are mainly modules of the ribosome (6 out of 12) and spliceosome (3 out of 12). Other large complexes include RNA polymerase, respiratory chain complex and the proteasome. The group of complexes with only 1 member are homo-dimers.

The CORUM database [26] is a manually annotated resource, containing, at the time of writing, 1679 protein complexes from 10 mammalian species, with a strong focus on human. Entries are based on specific publications, not high-throughput experiments. Table 1 lists Gene Ontology annotations for which CORUM deviates significantly from the rest of the genome. CORUM is enriched for nuclear proteins and contains a large number of transcriptional regulators. Conversely, extracellular and membrane proteins are underrepresented in the dataset. Figure 1 visually conveys an idea of the size distribution of this network of human complexes, as well as reflecting its highly interconnected nature. Relationships for 2080 proteins in 1109 human complexes were downloaded from the CORUM website [27]. 1975 Human Genome Nomenclature Committee (HGNC) identifiers [28], [29] for 2028 proteins could be mapped. Genomic coordinates for these gene identifiers were retrieved from EnsEMBL [30] *via* BioMART.

### Interaction and Complex Data

As an alternative to the manually compiled set of complexes in CORUM, an independent set of putative complexes was computationally derived from high-throughput protein interaction experiments by identifying highly connected clusters of proteins in an extended network of human protein interactions [31]. Data from three recent studies [32]–[34] were merged into one network. Interaction information was retrieved from IntAct [35], [36]. UniProt identifiers were mapped to HGNC identifiers using the cross references in the full UniProt entries. Clustering analysis was performed using the Markov clustering tool mcl [37] (parameter 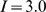). The alternative complex set was composed of all clusters with more than 3 components, containing 2325 unique genes.

### Set of Dosage Sensitive Genes

Dosage sensitive genes were extracted from the annotations of the Baylor College of Medicine Medical Genetics Laboratory 105k diagnostic Chromosomal Microarray (version 7) [38]. This post-natal screening tool comprises a manually compiled set of 146 genes known to be sensitive to chromosomal imbalances [39].

A separate set of genes overexpressed in cancer tissue was also used [40]. The dataset contains 2362 genes which are at least 4-fold overexpressed in brain (astrocytoma and glioblastoma), breast, colon, endometrium, kidney, liver, lung, ovary, prostate, skin, and thyroid cancers compared to healthy tissue of the same type.

### Expression Profiles

**Figure 2:**
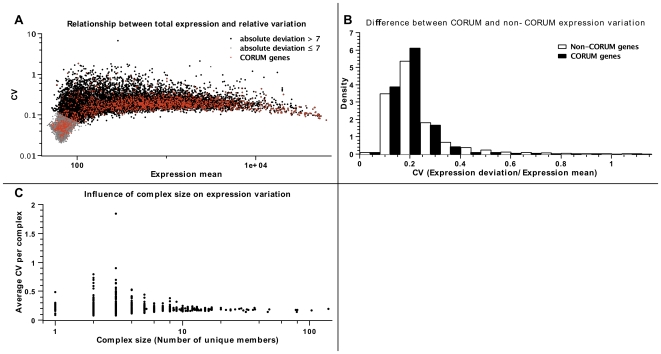
Coefficients of gene expression variation (CV), defined as standard deviation normalised to expression mean, vary for CORUM and non-CORUM genes. A) Effects of resolution and dynamic range of expression arrays on CVs. The measurable variation in gene expression is limited by the sensitivity of the employed array technology. Genes which are expressed at extremely low levels, or not expressed at all, cluster in the low expression/low CV region. Shown in grey are genes which were excluded from further calculations (standard deviation 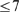). B) CORUM genes have significantly smaller CVs than non-CORUM genes. Outliers beyond 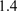 are not shown. C) Large CORUM complexes exhibit lower average CVs of their members.

Gene expression data were acquired from two independent sources: Expression data for 44760 probes applied to samples from 79 different tissue types were provided by GNF SymAtlas [41], [42]. Population-independently normalised expression data for 47293 probes applied to samples from lymphoblast cell lines of 270 HapMap individuals were provided by Stranger *et al.* [43], [44]. Probe identifiers were mapped to HGNC gene names through EnsEMBL BioMart. Probes which could not be mapped to a gene name were exluded from further analysis. The resulting matrices contained expression data for 17122 genes (HapMap set) and 15012 genes (tissue set), respectively. Due to technical limitations of the Illumina WG6 expression arrays used by Stranger *et al.*, there is a correlation between detectable expression variation and total expression strength (Figure 2A) for genes with low overall expression. Therefore, 6440 genes with an absolute population standard deviation 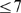 were removed from the dataset.

### Correlation Computation

As a measure of correlation between expression levels of two genes in different tissues/individuals, the Pearson product-moment correlation coefficient was employed. For two vectors 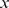 and 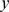 representing genes with 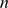 expression levels, the correlation 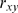 is given by
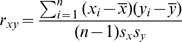
(1)where 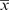 and 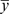 are the means and 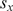 and 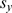 are the standard deviations of 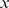 and 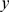, respectively. For complexes with more than 2 components, correlations for all 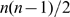 combinations of gene pairs were averaged.

### Copy-Number Variations

Chromosomal locations of variations relative to the NCBI36 human genome assembly were downloaded from the Database of Genomic Variants (DGV) [45], [46]. This data also contains information on number of individuals and gain/loss annotation per CNV. CNV locations and WGTP array hybridisation values for each HapMap individual were provided by Redon *et al.* [47], [48].

### Segmental Duplications

Segmental duplications of 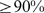 sequence identity and 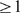 kilobase length were provided by the segmental duplication database [49], [50].

### Gene Ontology Analysis

Gene Ontology (GO) annotations from the GOA project [51] were mapped to HGNC identifiers through UniProt accessions. To reduce complexity, GO terms were integrated into GO-slim categories, as defined by the GOA project [52].

### Analysis of Selection Pressure

dN/dS values for human genes relative to mouse orthologs were acquired from EnsEmbl via BioMart. Only genes with a single unique ortholog in mouse were used in the analyses.

### Identification of Paralogs

In-species paralogs for 10755 HGNC gene identifiers were downloaded from EnsEmbl Compara via BioMart. The paralog prediction uses automatically generated maximum-likelihood phylogenetic trees. Details can be found at the EnsEMBL compara website.

### P-Values

Statistical significance of overlaps between gene sets was computed with Fisher's exact test (FET). The Mann-Whitney-U test (MWU) was employed to determine significance of differences between two distributions. In cases of multiple testing, Bonferroni correction was applied. All calculations were performed in R [53]. Significance of differences in dN/dS ratios was calculated by random resampling: For the null hypothesis, 1000 sets of genes with identical size as the test set were each created by randomly drawing without replacement from the complete gene set. P-Values were calculated as the probability of observing a result at least as extreme, given the normally distributed null model derived from the resampling.

## Results

### Effects of CNVs on Gene Expression

Association studies [43] have shown both *cis* and *trans* effects of copy-number variations (CNVs) on genes. However, there are few reports of a direct quantitative effect on expression levels for genes inside a specific CNV [54]. We therefore focused our attention on the relationship between copy-number variations and gene dosage. We combined gene expression data derived from lymphoblast cell lines of 270 HapMap individuals [43] with the CNV dataset of Redon *et al.* [47] on the same individuals.

**Figure 3:**
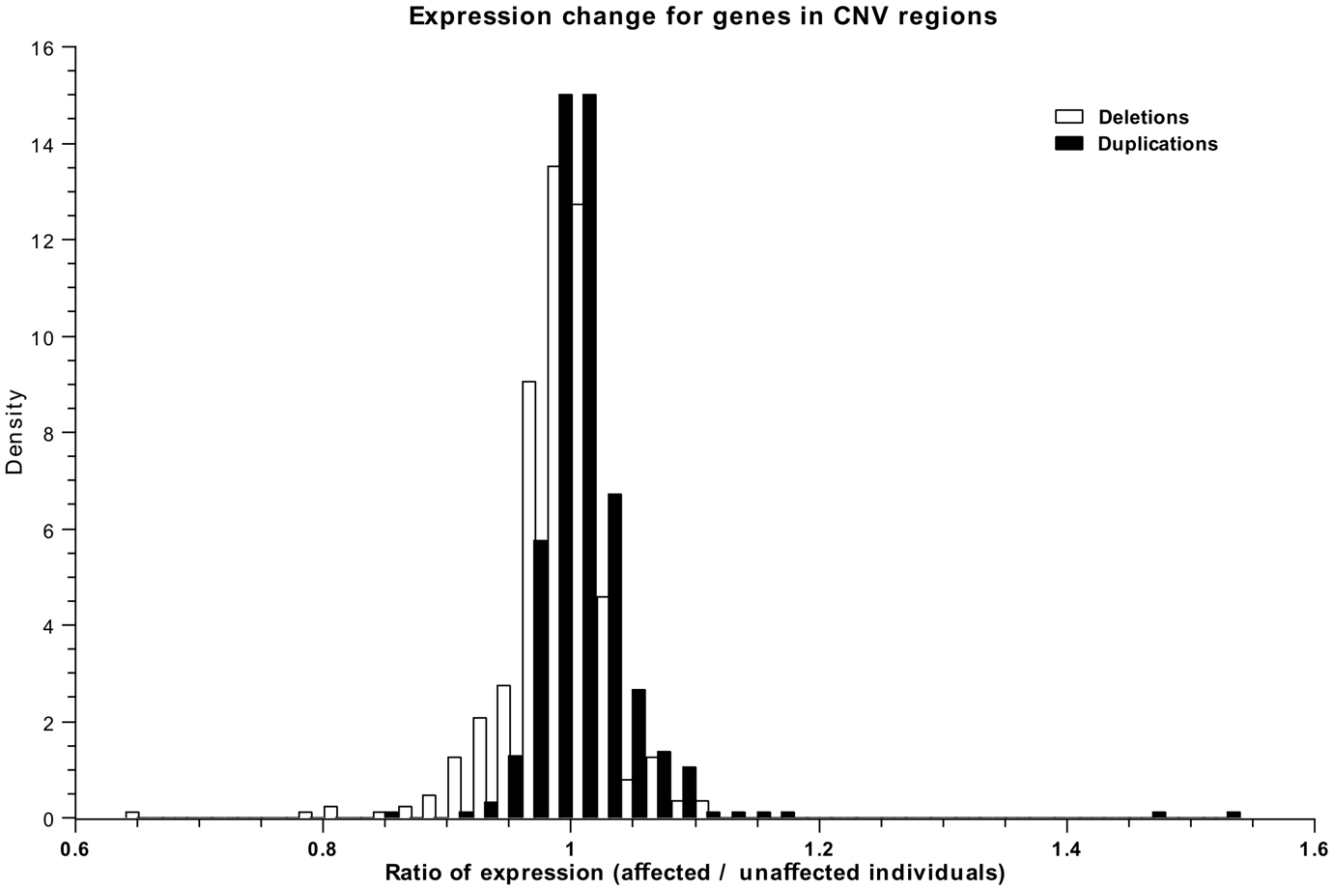
Difference between deletion (white) and duplication (black) variations in HapMap individuals. The histograms show the ratio of average expression levels between individuals with and without the CNV for all genes inside a CNV region. The shift between the two distributions is significantly larger than would be expected by chance (MWU: 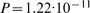).

We find that duplications and deletions have distinguishable profiles of expression ratios. The expression ratio is defined as the average expression of a gene in individuals with a CNV phenotype, divided by the average expression in wild-type individuals. Assuming a simple linear relationship between copy-number and expression level, one would expect a distribution with peaks at 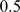, 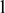 and 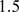, corresponding to a heterozygous deletion, balanced expression and heterozygous duplication, respectively. The observed distribution shown in Figure 3 reflects a more complex relationship.

The magnitude of the expression difference between CNV and wild type individuals is smaller and more continuous than expected. However, the location shift between the two distributions is highly significant (MWU: 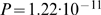). This indicates that deletions reduce gene expression, while duplications tend to increase expression. As mentioned in the Methods, sensitivity and dynamic range of the expression arrays could partly account for the observed noise, but we did not find a correlation between absolute gene expression level and ratio of expression difference for genes overlapping CNV regions (data not shown).

**Figure 4:**
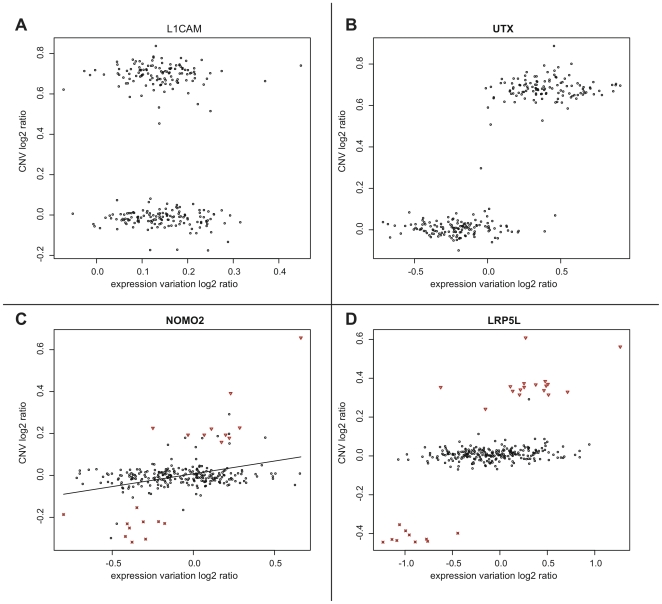
Ratio of WGTP array hybridisation intensity over relative expression level for four example genes. A) L1CAM and B) UTX. The increase in expression as a result of the copy-number increase in females is clearly visible for UTX which is known to escape X-inactivation. C) and D) Examples of autosomal genes with common CNV polymorphisms. Red crosses denote individuals in which a deletion phenotype has been called by Redon *et al.*, red triangles denote duplications. The plot highlights several potential false negatives with similar expression and hybridisation strength as the called deletions/duplications. Non-CNV related expression variation is substantial.

The expression ratio distribution reflects a summary over a wide range of individuals. To elucidate the effects of CNVs on gene expression on a per-individual basis, we plotted the logarithm of hybridisation strength on the genomic hybridization arrays relative to the reference individual (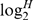) against the logarithm of expression, relative to the reference individual (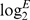). We find several examples of a linear relationship between copy-number and gene expression. As a positive control, we compared two X-chromosomal genes, one being inactivated (L1CAM, Figure 4A), the other being known to escape X-inactivation (UTX, Figure 4B). The latter exhibits a marked increase in expression in female individuals relative to the (male) reference individual. In contrast, L1CAM maintains equivalent expression levels due to inactivation of one gene copy.

Figures 4C and 4D show two examples of copy-number varied genes with induced dosage effects. Deletions and duplications have clearly distinguishable expression levels. Notably, though, the expression ratios of the deletion/duplication individuals overlap with the expression ratios of wild-type individuals. In other words, CNVs only partly account for the differences in expression between individuals, while a large portion of the variance must stem from other sources.

Furthermore, several individuals were not called as CNVs, despite similar 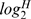 and 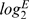 ratios in the analysed region as the identified CNV individuals. These putative false negatives will reduce the magnitude of expression ratios between CNV and wild-type individuals. Summarising these individual effects leads to the conclusion that duplications and deletions affect gene dosage, although they are not usually the primary sources of expression differences between individuals.

### Limited Expressional Noise of Protein-Complex Genes

**Figure 5:**
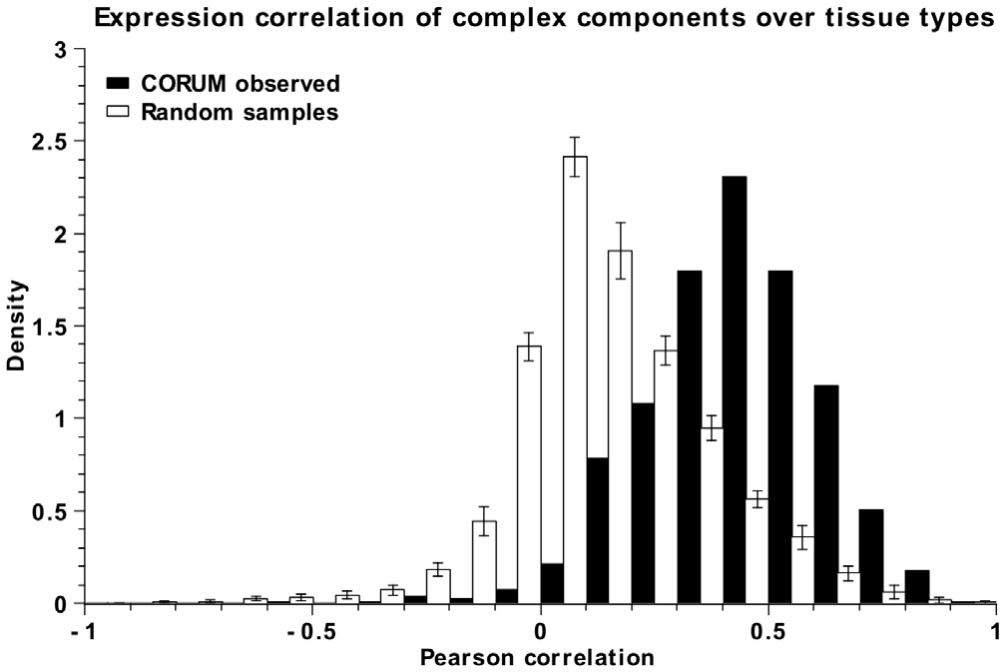
Distribution of average Pearson correlation coefficients between all members of known proteins complexes as defined in CORUM (black), and randomly sampled proteins (white, N = 10). Expression data was taken from the Human Gene Expression Atlas (see Methods).

It has previously been reported that expression levels of proteins within a complex are significantly more correlated across tissue types than would be expected by chance [16], [55]. Using both the expression from HapMap individuals mentioned above as well as a tissue-specific gene expression dataset, we verify that members of complexes from the CORUM database exhibit increased expression correlation (Figure 5).

In addition to that, the HapMap expression data allow us to perform a direct comparison of expression levels between individuals. We calculated coefficients of variation (CV), defined as the average variation in absolute expression levels per gene, normalised to the mean absolute expression level, see Figure 2. The CVs are significantly smaller for CORUM genes than for the rest of the genome (MWU: 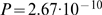). Interestingly, the average CV of genes within one complex decreases with the size of the complex, as shown in Figure 2C. We asserted that this effect is not a sampling artefact: Splitting all CORUM genes into sets with complexes of size 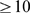 and size 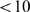 and comparing the distribution of CVs, we see that small complexes possess higher CVs (MWU: 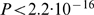). These results indicate that members of protein complexes are not just more likely to maintain relative expression levels between tissue types, but they are also more restricted as to their expression variation between individuals within the same tissue.

Axelsen *et al.* [40] compiled a list of 2362 genes which are overexpressed in various cancer tissues (see Methods). We speculated that these cancer related genes will be enriched for dosage sensitive genes whose overexpression in turn contributes to the disease phenotype. Consequentially, we find that CORUM is enriched for these cancer related genes (356 genes, FET: 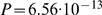). The fact that the tight regulation of expression of CORUM genes is disturbed in cancer tissue provides an interesting link between cancer, protein complexes and dosage sensitivity.

CORUM is a manually curated data source and thus prone to ascertainment bias. To ensure that these results are not biased by the composition of CORUM, we generated a separate dataset of putative protein complexes extracted from several high-throughput protein interaction detection experiments (see Methods). The clusters represent an alternative set of “complexes” composed of 2325 proteins, 505 of which are also contained in CORUM. The CV distribution difference between these highly interacting proteins and the rest of the genome is also skewed towards lower CVs (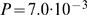). This suggests that highly connected proteins in general avoid imbalances in protein expression.

### Dosage Sensitive Genes and CNVs

We have shown above that protein complexes are under constraint to maintain their relative expression levels and exhibit limited expression variability between individuals. For our further analysis of dosage sensitivity, we also used an independently assembled set of 146 genes with known dosage-related disease phenotypes (see Methods). There is a significant overlap between CORUM and this set of dosage sensitive genes (32 genes, FET: 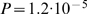), further supporting the link between dosage sensitivity and protein complexes. We henceforth use these two datasets as examples of dosage sensitivity.

As previously stated, we found that CNVs can affect the expression levels of genes they contain. We therefore hypothesised that a CNV that encompasses a gene which is part of a protein complex will be more likely to have a negative effect on fitness. As most available CNV data were derived from healthy individuals, we expect that genes encoding protein complexes will be underrepresented in CNV regions.

Out of 18534 protein coding genes for which both genomic locations and a unique gene name could be retrieved, 2311 genes are fully inside a CNV region. From 1975 proteins in the CORUM database, only 165 are found in a CNV region, significantly fewer than one would expect by chance (FET: 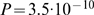). The set of automatically clustered complexes were also underrepresented in CNV regions (256 out of 2325 genes, 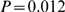). Lastly, both the set of 146 dosage sensitive genes (8 genes overlap, 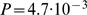) as well as the 2362 genes overexpressed in cancer (246 genes overlap, 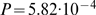) are unlikely to be contained in CNV regions.

Nguyen *et al.* as well as Cooper *et al.* reported a highly significant depletion of genes with the Gene Ontology (GO) category “binding” within CNV regions, but they do not comment further on this fact. We verified independently that “binding” is the second most underrepresented GO category after “intracellular” amongst genes in CNV regions. This lends further support to the hypothesis that dosage sensitivity due to protein complex membership has an influence of the composition of CNV regions.

We speculated that a negative fitness effect due to a copy-number variation will increase the likelihood of subsequent removal of that CNV from the gene pool. The CNVs that contain CORUM genes occur in significantly fewer individuals (MWU: 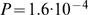) than non-CORUM genes, indicating that purifying selection has acted on some of the genes.

We also tested whether CORUM genes are underrepresented in gains compared to losses. Out of the 167 CORUM genes that overlap a CNV, 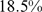 occur in a gain, compared to 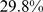 of non-CORUM genes. This significant difference in ratios (FET: 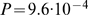) suggests that amongst copy-number varied genes, there is indeed a bias against duplications for genes in protein complexes, supporting the notion that stoichiometric imbalance has a negative effect on protein complexes.

### Compositional Bias of Copy-Number Varied Genes

Various compositional biases on genes in CNV regions have been described [1], [4]. Most notably, it has been reported that genes within CNV regions exhibit higher dN/dS than the rest of the genome. Is the observed low frequency of CORUM and other dosage sensitive genes in CNV regions merely a result of a bias against faster evolving genes? We verified that dN/dS ratios of genes within CNV regions were elevated compared to their mouse orthologs (Median: 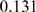, P-Value by resampling: 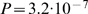). CORUM genes exhibit lower than expected dN/dS (Median: 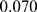, 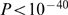). In contrast to non-complex genes, there is no significant difference in dN/dS between CORUM genes that overlap CNVs and those that do not. We therefore tested if there is a causal relationship between complex membership, low dN/dS and CNV overlap.

Like CORUM genes, the automatically clustered complexes also exhibited low dN/dS (Median 0.08, 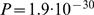). It has been argued that proteins with obligate interactions are under stronger selective pressure [56], which could explain the low dN/dS in both CORUM and the automatically clustered complexes. Interestingly, Cooper *et al.* showed that CNVs and segmental duplications (SDs) are of fundamentally similar nature and frequently overlap. We thus hypothesised that the reduction in negative selection within CNVs is related to the higher copy number of some genes which have been recently duplicated in a fixed SD. If we split the genes in CNV regions into those that overlap a SD and those that do not, we see that dN/dS ratios are highly significantly elevated in the genes that overlap SDs (MWU: 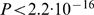), but not in the group outside SDs (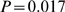).

Subsequently, we analysed the distribution of numbers of paralogs for human genes. We found that genes in CNV regions have significantly more paralogs than would be expected by chance (MWU, 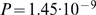),whereas genes from CORUM have significantly fewer (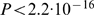). As with the evolutionary rate, the increase in numbers of paralogs is largely driven by CNVs that overlap SDs. Removing all genes inside SDs reduced the number of paralogs substantially (P-value reduced to from 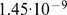 to 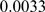). Conversely, the genes that are in both CNVs and SDs have significantly more paralogs than genes only found in CNV regions (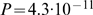). We conclude that the increase in dN/dS in CNV regions is driven by an increase in gene copy number and thus does not explain the underrepresentation of dosage sensitive genes in CNV regions.

If SDs are largely responsible for the increased dN/dS within CNVs and the increase in number of paralogs, can we still detect the underrepresentation of CORUM genes in CNVs that do not overlap a SD? We recalculated the contingency tables after removing all genes that overlap a SD. CORUM genes are still significantly underrepresented (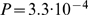), indicating that negative selective pressure not only affects regions of segmental duplication but also other types of CNVs.

## Discussion

### Protein Complexes Are Sensitive to Alterations in Gene Expression

Correlated gene expression of interacting proteins is a well known phenomenon, to the extent that correlation analysis is used to validate high-throughput protein interaction experiments [55]. Usually, expression data is gathered under diverse physiological conditions, *e.g.* at different stages of cell cycle. In our analysis, we have compared data from 79 different human tissue types. As expected, we observe strong correlation between the changes in gene expression for members of the same protein complex in different tissues. This observation hints at the importance of tightly regulated gene expression for the correct functioning of protein complexes.

However, it does not directly verify if the stoichiometry of complexes is under the same strong regulation. We therefore measured the variation in expression levels for interacting proteins in different HapMap individuals. Expressional noise of protein complexes has been analysed in yeast and fruit-fly [57], but the HapMap gene expression data allow the first systematic evaluation of protein complex expression in human. We find that genes in CORUM exhibit significantly smaller variation in expression than the rest of the genome. This is direct evidence that expression of complex genes is under tighter regulation than the rest of the genome. Furthermore, we see that genes in large complexes maintain particularly low expression variation. While we cannot rule out that this observation is due to functional constraints on the particular complexes, it does suggest that sensitivity to expressional noise is related to the number of subunits a complex maintains.

When we analysed the composition of genes in CNV regions, we made the curious observation that the small number of CORUM genes that overlap a CNV (165 genes in total) are biased towards deletions rather than duplications. If we assume that negative selection is acting on CNVs, the intuitive biological explanation for this phenomenon would be that CORUM genes are at least as sensitive to duplication than deletion, which in turn supports the concept that members of protein complexes are sensitive not just to under- but also to overexpression.

We made another observation that support this hypothesis. When comparing a manually curated set of dosage sensitive genes derived from the scientific literature, we found that a significantly larger than expected proportion of these genes were members of a protein complex as defined by the CORUM database. Taken together, these findings clearly indicate that stoichiometric fluctuations negatively affect protein complexes.

### CNVs Affect Expression Levels of Contained Genes

A key proposition that underpins our understanding of dosage sensitivity is that duplication or deletion of the genomic region containing a gene will result in a significant up- or downregulation of expression of the gene [58]. There have been previous reports of widespread expressional silencing of chromosomal amplifications [54]. In contrast, we observed lower average gene expression in deletion CNVs compared to duplication CNVs (Figure 3). It has to be noted, though, that these differences in expression are small for the majority of genes within a CNV. Furthermore, there are numerous cases where deletions seemingly result in increased expression and vice versa. Figures 4C and 4D exemplify how noisy the expression data for a gene can be, despite a visible expression difference between deletion and duplication genotypes. Sensitivity to detect expression differences at low concentration is not the main source of this variability in gene expression. Rather, we suspect there to be inherent fluctuations between the different cell lines used in the analysis [59]. Expressional noise alone does not explain that some CNVs seem not to affect gene expression at all. Rather, the inaccurate prediction of start and end coordinates of CNVs is likely to be largely responsible for the lack of correlation between CNVs and gene expression. Individuals with a CNV genotype falsely labelled as unaffected, or a gene erroneously placed inside a CNV, will skew the distribution of expression ratios.

We speculate, however, that there could also be a physiological explanation for the unexpectedly low change in gene expression upon copy-number variation. It is conceivable that the cell attempts to compensate changes in copy number on gene expression by *e.g.* increasing or decreasing transcription or modulating mRNA degradation. Such autosomal dosage compensation has been first observed in maize and *Drosophila* [60]–[62] and a general mechanism for dosage regulation has been proposed [63]. According to this theory, dosage balance is achieved through a network of regulatory genes which themselves are therefore dosage sensitive. The enrichment of CORUM for regulatory and transcription related functions might thus explain its sensitivity to copy-number variation and the low effect of CNVs on gene expression at the same time. With the arrival of new CNV datasets featuring improved breakpoint accuracy, it should become possible to better distinguish between false positive predictions and genes that are actually subject to dosage compensation. Subsequently, this will make it possible to determine the frequency of dosage compensation of copy-number varied genes.

### CNVs as the Source of Recent Duplications

It has been noted [4] that genes within CNV regions exhibit higher than expected dN/dS ratios, suggesting a relaxation of selective pressure. On the contrary, complex genes, dosage sensitive genes and highly connected genes in general, show very low dN/dS ratios, irrespective of whether they overlap CNVs or not. Stronger selective constraints in highly connected proteins have previously been attributed to functional constraints on the protein surface in order to maintain multiple binding sites [56].

Interestingly, we also show that genes in CNV regions have significantly more paralogs than expected by chance, while genes in protein complexes possess, on average, fewer paralogs [10]. This suggests that CNV regions have been hot-spots of large scale variation for a prolonged period of time, as it has also been shown that gene-rich CNV regions correspond well with regions of segmental duplications [1]. In fact, we found that those CNV regions that overlap segmental duplications are primarily (though not exclusively) responsible for the high number of paralogs.

Conversely, the reason for the increase in dN/dS in many genes within CNV regions could be attributed to their higher number of paralogous sequences. In fact, genes in CNVs overlapping segmental duplications are again primarily, but not exclusively, responsible for the elevated dN/dS ratios. These observations underline that CNV regions are a frequent source of gene duplicates which occasionally get fixed over the course of evolution and thus drive evolution of some gene families.

### Dosage Sensitivity and Negative Selection on CNVs

We observed that CNV regions are less likely to contain genes encoding protein complexes, as well as other dosage sensitive genes. Furthermore, CNVs which occur in multiple individuals and can thus be assumed to be older than unique CNVs are particularly depleted of CORUM genes. Hence, it appears that pressures on correct dosage limit the set of genes which can sustain variation in copy-number, even though the effect of CNVs on gene expression is not straightforward.

Dang *et al.* [64] reported that haploinsufficient genes are seldomly found between two regions of segmental duplication. Our results shed new light on this finding: It seems that dosage sensitive genes in general are biased against regions in which they are prone to suffer from copy-number variation. Segmental duplications are the most common source of such rearrangements, however we show that other CNVs not related to segmental duplications are also depleted of dosage sensitive genes. This indicates that negative selection is acting at the level of CNVs. Our findings offer a partial but consistent explanation for the biased composition of CNV regions. In addition to that, the correlation between dosage sensitivity and protein complex membership provides a convenient way to predict which genes are likely to be important in diseases which involve genomic rearrangements. The enrichment of CORUM for genes upregulated in cancer clearly hints towards this possibility. Future investigations should focus on the involvement of CNVs of putative dosage sensitive genes in cancer and complex diseases.
